# A Hierarchical SnO_2_@Ni_6_MnO_8_ Composite for High-Capacity Lithium-Ion Batteries

**DOI:** 10.3390/ma15248847

**Published:** 2022-12-11

**Authors:** Jiying Li, Jiawei Long, Tianli Han, Xirong Lin, Bai Sun, Shuguang Zhu, Jinjin Li, Jinyun Liu

**Affiliations:** 1 Key Laboratory of Functional Molecular Solids of the Ministry of Education, Anhui Provincial Engineering Laboratory for New-Energy Vehicle Battery Energy-Storage Materials, College of Chemistry and Materials Science, Anhui Normal University, Wuhu 241002, China; 2 National Key Laboratory of Science and Technology on Micro/Nano Fabrication, Department of Micro/Nano-Electronics, Shanghai Jiao Tong University, Shanghai 200240, China; 3 College of Environment and Energy Engineering, Anhui Jianzhu University, Hefei 230601, China

**Keywords:** lithium-ion batteries, anode, semiconductor, hierarchical structure, capacity

## Abstract

Semiconductor-based composites are potential anodes for Li-ion batteries, owing to their high theoretical capacity and low cost. However, low stability induced by large volumetric change in cycling restricts the applications of such composites. Here, a hierarchical SnO_2_@Ni_6_MnO_8_ composite comprising Ni_6_MnO_8_ nanoflakes growing on the surface of a three-dimensional (3D) SnO_2_ is developed by a hydrothermal synthesis method, achieving good electrochemical performance as a Li-ion battery anode. The composite provides spaces to buffer volume expansion, its hierarchical profile benefits the fast transport of Li^+^ ions and electrons, and the Ni_6_MnO_8_ coating on SnO_2_ improves conductivity. Compared to SnO_2_, the Ni_6_MnO_8_ coating significantly enhances the discharge capacity and stability. The SnO_2_@Ni_6_MnO_8_ anode displays 1030 mAh g^−1^ at 0.1 A g^−1^ and exhibits 800 mAh g^−1^ under 0.5 A g^−1^, along with high Coulombic efficiency of 95%. Furthermore, stable rate performance can be achieved, indicating promising applications.

## 1. Introduction

Given the numerous applications of lithium-ion (Li-ion) batteries in our daily lives, the development of high-performance Li-ion batteries has attracted increasing attention, as they are important sources of clean and renewable energy. The demands of next-generation Li-ion batteries include sufficient energy density and improved safety and cycle life. However, at present, graphite anodes with a low theoretical capacity are dominant, restricting the development of emerging Li-ion batteries [1,2,3,4]. In addition, lithium dendrite formed under high current density is associated with safety problems. Therefore, commercial graphite anodes cannot satisfy current demands. In recent years, researchers have developed several promising anode materials with increased capacity and safety with potential to replace graphite to improve performance [5,6]. Many nanostructured anodes have been reported, such as metal oxides (e.g., MnO_2_, GeO_2_, Co_3_O_4_, Fe_2_O_3_, SnO_2_, etc.) [7,8,9,10] and alloying–dealloying mechanism-based anodes (e.g., Si, Si/SiC, Si/C/SiC, Si/Ge, etc.) [11,12,13], many of which exhibit satisfactory capacity and rate performance compared to graphite-based anodes, given their high theoretical capacities. The application of such anodes is still limited by defects, such as large volume change and poor conductivity [14,15,16,17,18,19,20,21].

SnO_2_ has been widely considered one of the most promising anodes among metal dioxides in recent years, owing to its abundance, environmental friendliness, and Li-ion storage performance. Nevertheless, its theoretical capacity (782 mAh g^−1^) and the volume change of SnO_2_ in lithiation/delithiation lead to a rapid decrease in capacity. The low conductivity of SnO_2_ also leads to poor rate performance and low capacity retention. Furthermore, large volume change leads to electrode pulverization [22,23,24]. Liu et al. reported a carbon-coating-layered SnO_2_ hollow sphere, which evenly coated SnO_2_ with a carbon nanolayer [25]. The hollow structure and carbon buffer layer improved the capacity significantly. Zhou et al. synthesized a hollow SnO_2_@C combining SnO_2_ with carbon. The hollow sphere ensured structural stability during long-term charge and discharge, exhibiting stability of 1628 mAh g^−1^ under 0.1 A g^−1^ [26]. Liu and colleagues reported a graphene-mesoporous SnO_2_ combining SnO_2_ with conductive graphene, which exhibited improved conductivity [27].

In addition, bimetal oxides such as CuCo_2_O_4_, ZnFe_2_O_4_, CoMn_2_O_4_, ZnCo_2_O_4_, and ZnMn_2_O_4_ exhibit satisfactory electronic conductivity and reversible capacity, which may be caused by the heterovalent cations and corresponding general redox reactions [28,29]. Zhang et al. prepared a yolk–shell CoMn_2_O_4_ microsphere using a carbon template with a solvothermal method [30]. The yolk–shell structures of CoMn_2_O_4_ microspheres buffered the volumetric change in Li^+^ insertion/extraction, thereby reducing electrochemical pulverization. CoMn_2_O_4_-based anode exhibited 1643 mAh g^−1^ at 0.1 A g^−1^. Ren et al. presented ZnCo_2_O_4_@reduced graphene oxide nanocomposites; ZnCo_2_O_4_ nanoparticles were anchored uniformly and compactly on reduced graphene oxide. High specific surface area and mesoporous structure provided a large contact area with an electrolyte and shortened the transferring pathway of the Li^+^ ions [31]. Chen et al. prepared a hollow panpipe-like ZnMn_2_O_4_ nanocomposite, which was conducive to the penetration of the electrolyte during the charge/discharge process [32]. However, the intercalation/deintercalation of Li^+^ in those composites would cause considerable volume change during cycling, resulting in serious agglomeration of the materials and the exfoliation of the electrode materials from the collector, rapidly reducing the performance. Engineering optimal structures to efficiently accommodate the volume change has become an important strategy to improve Li storage properties [33,34].

**Figure 1:**
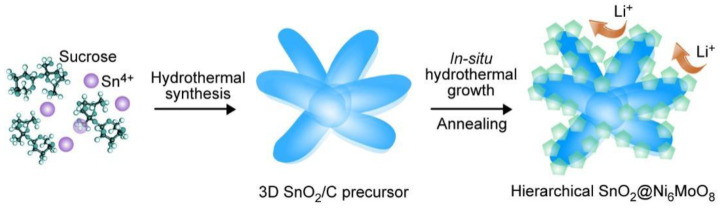
Preparation of a hierarchical SnO_2_@Ni_6_MnO_8_ composite.

Here, we developed a hierarchical SnO_2_@Ni_6_MnO_8_ composite comprising Ni_6_MnO_8_ nanoflakes growing on the surface of a three-dimensional (3D) SnO_2_, as illustrated in Figure 1. The SnO_2_@Ni_6_MnO_8_ composite was prepared through a two-step hydrothermal method. After calcination, hollow SnO_2_ was formed, providing space for the penetration of electrolytes, benefitting Li^+^ ion diffusion and accommodating the volumetric change during cycling. The special structure and composition of composites have rarely been reported. Composite-based anodes achieve positive electrochemical performance. The capacity remains 1030 mAh g^−1^ after cycling 50 times under 0.1 A g^−1^ and 800 mAh g^−1^ under 0.5 A g^−1^, in addition to high Coulombic efficiency exceeding 95% and recoverable rate performance.

## 2. Experimental

### 2.1. Preparation of 3D SnO_2_/C Precursor

First, 20 mmol sucrose was dissolved in 40 mL of deionized water. Then, 10 mmol SnCl_4_·5H_2_O (Aladdin Co. Ltd., Beijing, China) was added to the solution with continuous stirring. After complete dissolution, it was transferred into a PTFE-lined stainless-steel reactor and reacted at 180 °C for 6 h. Then, the obtained precipitate was washed with water and ethanol alternately by centrifugation, then dried in an oven.

### 2.2. Preparation of SnO_2_@Ni_6_MnO_8_ Composite

An amount of 0.1 g of the SnO_2_/C precursor was ultrasonically dispersed in 40 mL of deionized water. Then, 300 mg of Ni(NO_3_)_2_·6H_2_O, 530 mg of Mn(NO_3_)_2_·4H_2_O, 140 mg of hexamethylenetetramine, and 29 mg of trisodium citrate dihydrate were subsequently added into the above solution under ultrasonic treatment. The solution was placed in the reactor at 140 °C for 6 h. Then, it was taken out and cooled. The sample was washed and dried at 60 °C and annealed at 500 °C for 1 h in air with a heating rate of 5 °C per min.

### 2.3. Characterization

The samples were characterized on a Bruker D8 X-ray diffractometer (XRD), a Hitachi HT-7700 transmission electron microscope (TEM), an S-8100 scanning electron microscope (SEM), a MICROMERITICS ASAP 2460 Brunauer–Emmett–Teller (BET) instrument, and an ESCALAB 250 X-ray photoelectron spectroscope (XPS).

### 2.4. Electrochemical Measurement

A coin cell system was used to measure electrochemical performance. First, 3D SnO_2_@Ni_6_MnO_8_, carbon black, and carboxymethyl cellulose were mixed (mass ratio = 8:1:1) and coated on Cu foil. After drying at 60 °C in a vacuum overnight, the sample was cut into small discs. 2032-typed cells were assembled by using a glove box (Mikrouna, Super 1220/750/900). Li foil was used as the counter electrode, and the Celgard polypropylene film was used as a separator. Electrolytes contained 1 M LiPF_6_ in ethyl carbonate and diethyl carbonate (volume ratio = 1:1). Galvanostatic charge–discharge was tested on a measuring system (Netware, CT-4008).

## 3. Results and Discussion

**Figure 2:**
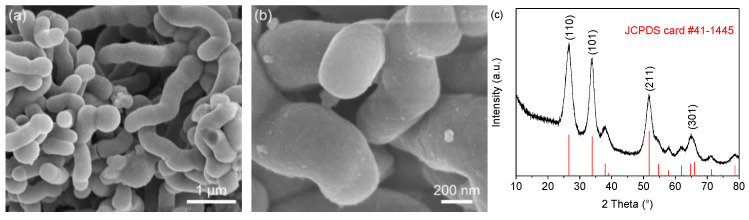

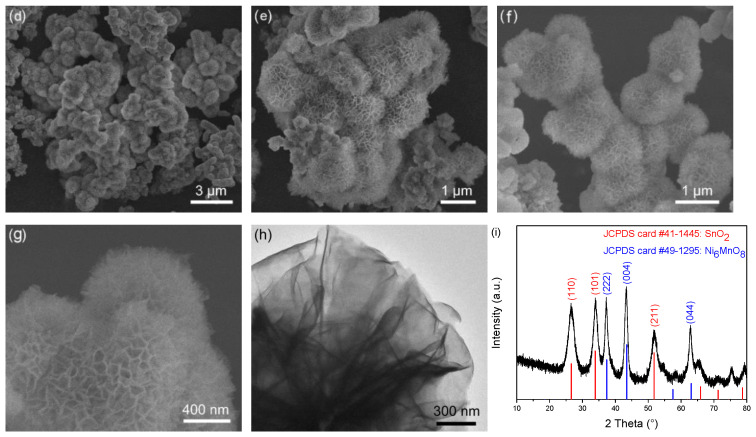
(**a**,**b**) SEM images and (**c**) XRD pattern of SnO_2_/C precursor. SEM images of SnO_2_@Ni_6_MnO_8_ precursor (**d**,**e**) before and (**f**,**g**) after annealing. (**h**) TEM image of the Ni_6_MnO_8_ nanoflakes on the surface of SnO_2_. (**i**) XRD pattern of the SnO_2_@Ni_6_MnO_8_ composite.

Figure 2a,b displays the SEM images of the 3D SnO_2_/C precursor, which exhibits a coral-like structure with a diameter of 400–500 nm. The XRD pattern of SnO_2_/C precursor is presented in Figure 2c. Four diffraction peaks were detected at 26.6°, 33.9°, 51.8°, and 65.9°, corresponding to the (110), (101), (211), and (301) planes of tetragonal SnO_2_ (JCPDS card #41–1445), respectively [35,36]. Figure 2d,e show the SnO_2_@Ni_6_MnO_8_ after in situ growth of Ni_6_MnO_8_ on SnO_2_/C precursor. The surface of SnO_2_/C is completely coated by dense Ni_6_MnO_8_ nanoflakes. The 3D profile is well-maintained, indicating a robust structure.

**Figure 3:**
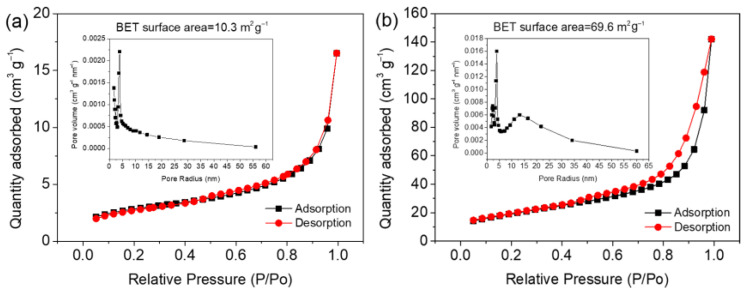
N_2_ adsorption–desorption isothermal curves of (**a**) SnO_2_@C and (**b**) SnO_2_@Ni_6_MnO_8_. Insert shows each pore distribution.

Figure 2f,g are SEM images of SnO_2_@Ni_6_MnO_8_ calcined at 500 °C. The morphology remains unchanged compared to that before heat treatment. Dense nanoflakes are coated on the surface without breaking. Figure 2h shows a TEM image of the composite. Because the tubular structure is covered by thick and dense nanoflakes, it is difficult to observe the SnO_2_ inside. The TEM image shows thin nanoflakes, which are beneficial in terms of providing large active sites for reaction with Li^+^ ions in Li-ion batteries. The XRD pattern of SnO_2_@Ni_6_MnO_8_ is displayed in Figure 2i. Compared with the diffraction profile shown in Figure 2c, diffraction peaks at 37.3°, 43.4°, and 63.1° are observed, corresponding to the (222), (004), and (044) planes of Ni_6_MnO_8_ (JCPDS card #49–1295), respectively, which verify a secondary growth. The BET measurements of SnO_2_@C and SnO_2_@Ni_6_MnO_8_ are shown in Figure 3. Owing to the coating of dense Ni_6_MnO_8_ nanoflakes, the BET surface area of SnO_2_@Ni_6_MnO_8_ is as high as 69.6 cm^2^ g^−1^ compared to SnO_2_@C (10.3 m^2^ g^−1^). The inserts show that pore distributions of SnO_2_@C and SnO_2_@Ni_6_MnO_8_ dominate at about 3 nm and 15 nm. The increased surface area and large pore volume promotes electrolyte penetration.

**Figure 4:**
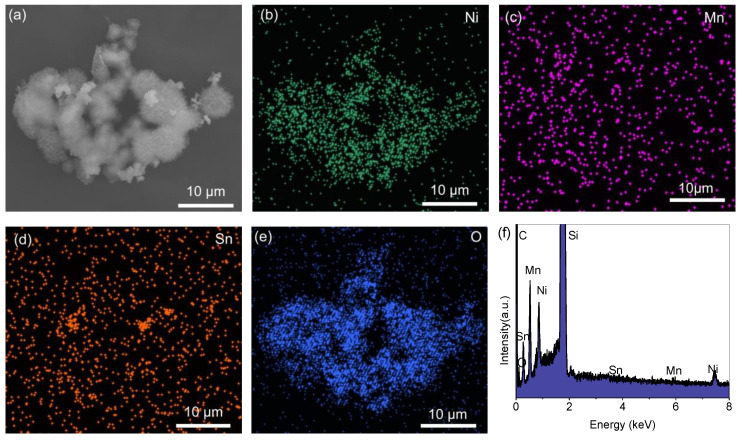
(**a**) SEM and corresponding mapping images of (**b**) Ni, (**c**) Mn, (**d**) Sn, and (**e**) O. (**f**) EDS spectrum.

An SEM image of SnO_2_@Ni_6_MnO_8_ and the elemental distribution are displayed in Figure 4. Sn, Ni, Mn, and O elements are evenly distributed throughout the composite. The EDS spectrum shown in Figure 4f confirms the presence of Sn, O, Mn, and Ni. The signal of Si is ascribed to the substrate used for measurement [37]. The profiles of Mn and Sn do not match well with the profile, which could be caused by the covering of signals by Ni and O, as has previously been reported in some surface-coated composites [38].

**Figure 5:**
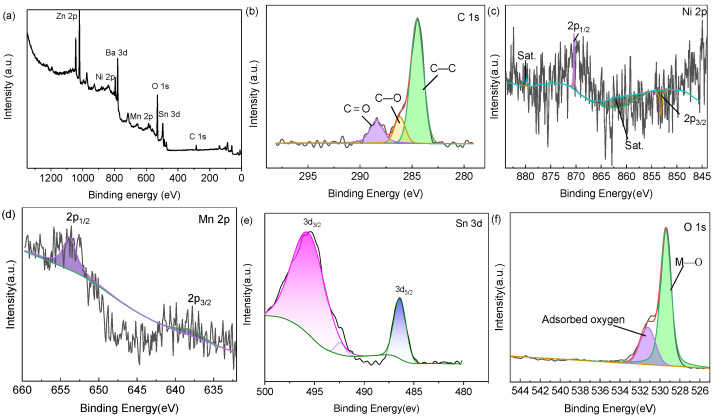
XPS spectra of SnO_2_@Ni_6_MnO_8_ composite: (**a**) survey spectrum; (**b**) C 1s; (**c**) Ni 2p; (**d**) Mn 2p; (**e**) Sn 3d; and (**f**) O 1s.

The XPS spectra of SnO_2_@Ni_6_MnO_8_ were measured. The coexistence of Mn, Ni, C, O, and Sn is verified by the XPS survey spectrum (Figure 5a). The XPS spectrum of C 1s (Figure 5b) is characterized by three peaks located at 284.4, 286.2, and 288.4 eV and indexed to C—C, O—C, and C═O groups, respectively [39]. The Ni 2p spectrum shows four peaks at 853.4, 861.5, 870.4, and 879.9 eV, corresponding to Ni^2+^ (Figure 5c). Figure 5d displays the Mn 2p peaks centered at 637.7 and 653.7eV, which are ascribed to Mn 2p_3/2_ and Mn 2p_1/2_, respectively. The Sn 3d spectrum (Figure 5e) verifies the existence of Sn 3d_3/2_ and Sn 3d_5/2_, confirming Sn^4+^ of SnO_2_ [27]. Some shifts of Sn elements occurred, which may be ascribed to the impact of the Ni_6_MnO_8_ coating on the surface of SnO_2_ [40]. In the O 1s spectrum (Figure 5f), 529.4 eV is associated with metal–oxygen bonds, whereas the other peaks are attributed to the lattice oxygen of Ni_6_MnO_8_ (530.9 eV) and surface-adsorbed oxygen (531.8 eV) [33,41,42]. Furthermore, the atomic percentages of Sn, Ni, Mn, and O are 1.46%, 0.99%, 1.72%, and 71.49%, respectively.

**Figure 6:**
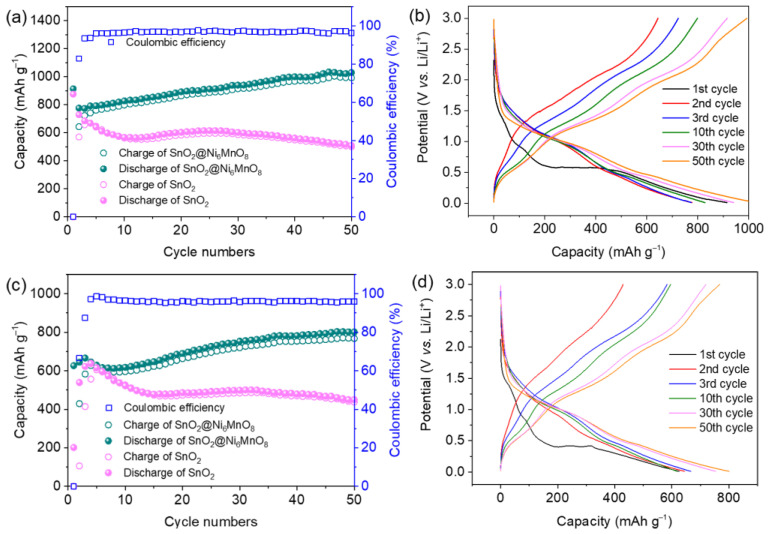
(**a**) Cycling performance of 3D SnO_2_@Ni_6_MnO_8_ and SnO_2_ anodes under 0.1 A g^−1^. (**b**) Corresponding charge–discharge profiles of SnO_2_@Ni_6_MnO_8_. (**c**) Capacity and (**d**) charge–discharge profiles under 0.5 A g^−1^.

The electrochemical properties of 3D SnO_2_@Ni_6_MnO_8_ nanocomposite and SnO_2_ were measured from 0.01 to 3.0 V. The cycling performance at a current density of 0.1 A g^−1^ is presented in Figure 6a. The SnO_2_@Ni_6_MnO_8_ anode shows a high specific capacity of 914 mAh g^−1^. After 50 cycles, the SnO_2_@Ni_6_MnO_8_ anode maintains a reversible capacity of 1030 mAh g^−1^, and its Coulombic efficiency exceeds 96%. The first-cycle capacity of SnO_2_ is 876 mAh g^−1^. However, the capacity decreases rapidly to 509 mAh g^−1^ after the 50th cycle, indicating that the Ni_6_MnO_8_ coating considerably enhances the capacity and stability. The slight increase in capacity is ascribed to the activation of the nanoscale composite. Figure 6b shows corresponding charge–discharge profiles of the SnO_2_@Ni_6_MnO_8_ anode. There are two plateaus on the discharge curves, which correspond to Li^+^ ion insertion in SnO_2_@Ni_6_MnO_8_. In the charging process, there are three plateaus at around 0.5–1.0 V, 1.2–2.0 V, and 2.0–3.0 V, which are assigned to Li^+^ ion extraction. Figure 6c shows the cycling performance at 0.5 A g^−1^. After cycling 50 times, the SnO_2_ anode shows a low capacity of 437 mAh g^−1^. In contrast, the capacity of SnO_2_@Ni_6_MnO_8_ is maintains as high as 800 mAh g^−1^, with a Coulombic efficiency of about 95%, indicating a sufficient reversibility. Figure 6d shows the cycling curves with a similar shape to those presented in Figure 6b, representing the same electrochemical behaviors at a relatively high current density. Compared to some other reported anodes, the SnO_2_@Ni_6_MnO_8_ anode exhibits a competitive performance, as shown in Table 1.

**Figure 7:**
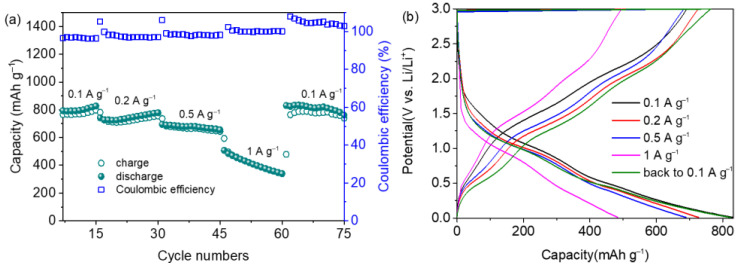
(**a**) Rate performance of the SnO_2_@Ni_6_MnO_8_ anode. (**b**) Corresponding charge–discharge curves.

Figure 7a,b show the rate performance of the 3D SnO_2_@Ni_6_MnO_8_ nanocomposite. Figure 7a shows that the capacities are 819, 775, 655, and 339 mAh g^−1^ at 0.1, 0.2, 0.5, and 1.0 A g^−1^, respectively. Once the rate returns to 0.1 A g^−1^, the capacity recovers to 760 mAh g^−1^. The galvanostatic charge–discharge curve for each current density with a similar profile is shown in Figure 7b, indicating satisfactory reversibility. The excellent rate performance and cyclic reversibility of the SnO_2_@Ni_6_MnO_8_ composite are attributed to the hierarchical morphology with nanoflakes growing in situ on a 3D structure, which provides efficient space to alleviate the volumetric expansion and enable rapid transport of electrons and ions.

**Figure 8:**
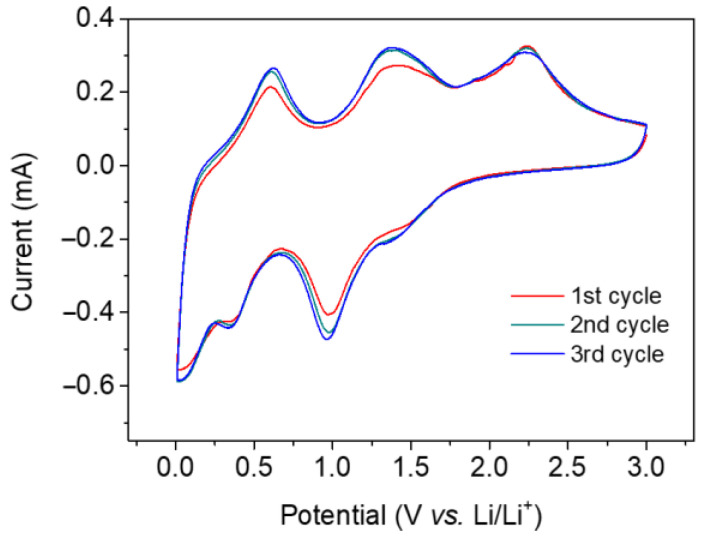
Cyclic voltammetry (CV) curves for Na_6_MnO_8_ at a scanning rate of 0.1 mV g^−1^.

**Figure 9:**
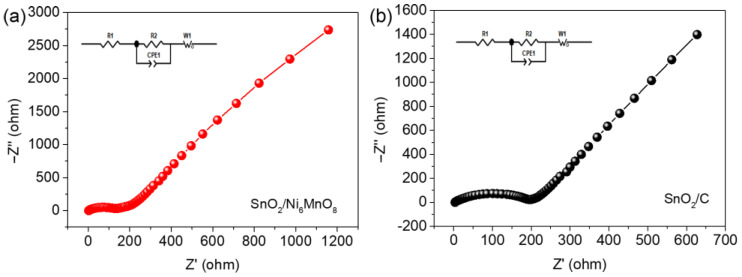
Nyquist plots for the (**a**) SnO_2_/Ni_6_MnO_8_ and (**b**) SnO_2_/C anodes.

CV curves are shown in Figure 8. There are three peaks in the cathodic process at 0.34, 0.96, and 1.39 V, indicating Li^+^ ion insertion in the SnO_2_/Ni_6_MnO_8_ anode. In contrast, the peaks at 0.61, 1.38, and 2.31 V are assigned to the extraction of Li^+^, in accordance with the charge–discharge curves shown in Figure 6 and Figure 7. The Nyquist plots presented in Figure 9 show the charge transfer resistances for SnO_2_/Ni_6_MnO_8_ and SnO_2_/C anodes. The fitting values before and after cycling are about 130 and 200 Ω, respectively, indicating an improved conductivity after coating with Ni_6_MnO_8_. An interesting line of future research would be to obtain the diffusion coefficient through EIS spectra [43].

**Table 1:** Comparison of electrochemical performance of SnO_2_ anodes.

Composite	Preparation Method	Cycling Rate (A g^−1^)	Cycle Number	Capacity (mAh g^−1^)	Ref.
SnO_2_@PANI	Electrochemical deposition	0.1	50	440	[44]
C@Sn–SnO_2_/CNT	Hydrothermal method and chemical vapor deposition	0.5	100	733	[45]
NiMoO_4_/SnO_2_/rGO	Hydrothermal method	0.5	100	634	[46]
SnO_2_@C-F	Balling method	0.2	100	821	[47]
RHPC/SnO_2_	Melt–impregnation method	0.1	50	550	[48]
SnO_2_/EG	Solvothermal method	0.5	500	262	[49]
SnO_2_@C	Acid etching method	0.1	150	745	[50]
SnO_2_@C/MWCNTs-LiF	Spray-drying method	0.1	100	483	[51]
SnO_2_/GNP	Microwave irradiation	0.1	100	745	[52]
SnO_2_@C	Templated method	0.2	100	786	[53]
SnO_2_@SnS_2_	Hydrothermal method	0.1	50	328	[54]
SnO_2_/TiO_2_	Spray drying and calcination	0.5	40	483	[55]
SnO_2_@Ni_6_MnO_8_	Hydrothermal method	0.1	50	1030	Thiswork
		0.5	50	800	

## 4. Conclusions

In summary, a hierarchical SnO_2_@Ni_6_MnO_8_ composite was developed for a secondary battery anode with satisfactory Li-storage performance by the hydrothermal method. The presented approach is controllable and simple; however, we expect that a higher-yield preparation can be achieved in the future. The 3D structure provides a large space to buffer volumetric change during charge and discharge. The hierarchical surface shortens the transferring distance of Li^+^ ions and electrons, and the Ni_6_MnO_8_ coating on SnO_2_ improves conductivity. Compared to SnO_2_, the coating of Ni_6_MnO_8_ considerably enhances the discharge capacity and stability of the anode. The capacities of SnO_2_@Ni_6_MnO_8_ anodes remain 1030 and 800 mAh g^−1^ after 50 cycles at 0.1 and 0.5 A g^−1^, respectively, and the Coulombic efficiency continues to exceed 95%. Moreover, a high and reversible rate performance is also achieved. We believe that the 3D structure presented here and the high electrochemical performance opportunities to engineer emerging composites for secondary battery systems. However, potential challenges should also be considered in future investigations, such as extending the applicable materials, reducing the cost of reagents and steps during synthesis, avoiding the toxicity, etc.

## Data Availability

Not applicable.
